# Supplementary material for: CT-optimal touch and chronic pain experience in Parkinson’s Disease; An intervention study
Source: PLoS One. 2024 Feb 23;19(2):e0298345. doi: 10.1371/journal.pone.0298345 (PMC10890780; doi:10.1371/journal.pone.0298345)
Supplement: S1 File — (DOCX) [file pone.0298345.s001.docx]

**Supplementary material File** Manuscript CT-optimal touch and chronic pain experience in Parkinson’s Disease; An intervention study

**Table S1. Descriptive statistics.**

| Variables | Mean (SD) | Possible range | N |
| --- | --- | --- | --- |
| Age | 64.41 (18.45) | 18-100 | 17 |
| Gender:  Male/Female | n.a. |  | 10/7 |
| Type of pain:  Musculoskeletal pain  Radicular pain | n.a. |  | 10  7 |
| Pain Intensity Score | 5.85 (1.55) | 0-10 | 17 |
| KPDPS total score | 38.06 (15.43) | 0-168 | 17 |
| QRI total score  Participant  Partner | 16.06 (1.95)  15.38 (1.80) | 6 – 24  6 – 24 | 16^  16^ |

*n.a.= not applicable
^= data of two participants and partners are missing.

**Table S2. Mean-difference and p-values for CT-optimal touch and CT non-optimal touch for the different timepoints.**

|  | CT-optimal touch | | CT non-optimal touch | |
| --- | --- | --- | --- | --- |
|  | Mean difference | *p* | Mean difference | *p* |
| 5min – 0min | -.35 | .005* | -.25 | .003* |
| 10min – 0min | -.66 | <.001* | -.50 | <.001* |
| 15min – 0min | -.82 | <.001* | -.52 | <.001* |
| 10min – 5min | -.31 | .001* | -.24 | .009* |
| 15min – 5min | -.47 | <.001* | -.27 | .013* |
| 15min – 10min | -.16 | .079 | -.03 | 1.00 |

*p<.05.
