# Supplementary material for: CT-optimal touch and chronic pain experience in Parkinson’s Disease; An intervention study
Source: PLoS One. 2024 Feb 23;19(2):e0298345. doi: 10.1371/journal.pone.0298345 (PMC10890780; doi:10.1371/journal.pone.0298345)
Supplement: Attachment — Submitted filename: Response to reviewers v2.docx [file pone.0298345.s003.docx]

**Response to reviewers**

Dear Julian Packheiser,

First of all, we would like to thank the editor and reviewers for the opportunity to address these minor revisions. These extra valuable comments have improved the manuscript even more. We sincerely hope that it now meets the criteria for publication in PLOSone. Below, in red we addressed every point from the reviewers. The line numbers refer to the manuscript with track changes.

Thank you again for considering our manuscript and we would like to wish you a Merry Christmas and a Happy New Year.

Kind regards also on behalf of the co-authors,

Larissa Meijer

Journal Requirements:

Please review your reference list to ensure that it is complete and correct. If you have cited papers that have been retracted, please include the rationale for doing so in the manuscript text, or remove these references and replace them with relevant current references. Any changes to the reference list should be mentioned in the rebuttal letter that accompanies your revised manuscript. If you need to cite a retracted article, indicate the article’s retracted status in the References list and also include a citation and full reference for the retraction notice.

The reference list has been checked and is correct, no adjustments have been made.

[Note: HTML markup is below. Please do not edit.]

Reviewers' comments:

Reviewer's Responses to Questions

**Comments to the Author**

1. If the authors have adequately addressed your comments raised in a previous round of review and you feel that this manuscript is now acceptable for publication, you may indicate that here to bypass the “Comments to the Author” section, enter your conflict of interest statement in the “Confidential to Editor” section, and submit your "Accept" recommendation.

Reviewer #1: (No Response)

Reviewer #2: All comments have been addressed

2. Is the manuscript technically sound, and do the data support the conclusions?

The manuscript must describe a technically sound piece of scientific research with data that supports the conclusions. Experiments must have been conducted rigorously, with appropriate controls, replication, and sample sizes. The conclusions must be drawn appropriately based on the data presented.

Reviewer #1: Partly

Reviewer #2: Yes

3. Has the statistical analysis been performed appropriately and rigorously?

Reviewer #1: Yes

Reviewer #2: Yes

4. Have the authors made all data underlying the findings in their manuscript fully available?

The [PLOS Data policy](http://www.plosone.org/static/policies.action#sharing) requires authors to make all data underlying the findings described in their manuscript fully available without restriction, with rare exception (please refer to the Data Availability Statement in the manuscript PDF file). The data should be provided as part of the manuscript or its supporting information, or deposited to a public repository. For example, in addition to summary statistics, the data points behind means, medians and variance measures should be available. If there are restrictions on publicly sharing data—e.g. participant privacy or use of data from a third party—those must be specified.

Reviewer #1: Yes

Reviewer #2: No

5. Is the manuscript presented in an intelligible fashion and written in standard English?

PLOS ONE does not copyedit accepted manuscripts, so the language in submitted articles must be clear, correct, and unambiguous. Any typographical or grammatical errors should be corrected at revision, so please note any specific errors here.

Reviewer #1: Yes

Reviewer #2: Yes

6. Review Comments to the Author

Please use the space provided to explain your answers to the questions above. You may also include additional comments for the author, including concerns about dual publication, research ethics, or publication ethics. (Please upload your review as an attachment if it exceeds 20,000 characters)

Reviewer #1: The authors present a fine revision of their manuscript and have well answered to my questions and comments. I only have a few minor suggestions and a more important one for interpreting the results.

Abstract: I think it should read “treated with analgesics” (not analgesia)

This is indeed wrong, we changed it to analgesics at line number 13.

Line 67: “appreciation” means recognizing the good qualities of something (Oxford dictionary). I don’t think this is what is meant here.

We changed this sentence to ‘which are important for the affective aspects of pain experience’ at line numbers 61-62.

Line 108: explain what “Gorilla” is when it is mentioned for the first time.

We added the following ‘’ Gorilla (an online survey tool)… “ at line number 110.

Figure 4 and 5: Say in the caption what the line shows (mean values I assume).

This has been added to the figure captions (see Figure file).

Line 329. There is no “S2 Table”. Do you mean “Table 2”?

We were referring to the S2 Table. I have checked the revised supplementary File as a supporting information file, the S2 Table is available there. This has been done according to the submission guidelines from PLOSone.

Line 380 ff. “an immediate effect of touch on chronic pain experience was also investigated. Here, the results showed that CT-optimal touch reduces the chronic pain experience significantly more compared to CT non-optimal touch”. This appears to be the interpretation of the results in lines 324-329. However, this interpretation does not seem justified. Similar to a pain reduction of CT-optimal touch, namely a significant difference between 0 and 5 minutes, and between 0 and 10, there is also a significant difference for CT-non-optimal touch between 0 and 5 minutes, and between 0 and 10. Thus, there is also a short-time pain-reducing effect of CT-non-optimal touch.

The only difference between CT-optimal and non-optimal touch is that for CT-optimal touch, there is an additional significant difference for the time interval between 5 and 10 minutes, which is not present for CT-non-optimal touch. For the other four intervals, CT and non-CT touch yield similar results. Thus, the interpretation that CT-touch is more efficient than non-CT-touch for short-term pain relief is on shaky grounds as it is based on just this one difference (one out of five). Consequently, it should be amended in both the discussion and the abstract. The conclusion appears to rather be that CT and non-CT touch are equally effective in short- as well as long-term pain reduction, which is perfectly fine. This would also fit to the findings from studies showing that even mere partner presence without touch can reduce pain (e.g.; Duschek et al, Scand J Pain 2019). I suggest you consider that it might be the support communicated through touch (or other means) that is crucial for pain amelioration, and not the type of touch itself. I leave this to the authors, but it might be interesting to discuss this aspect. Personally, I think this would be more beneficial to the reader than exaggerating the small CT effects.

We fully agree that this is effect is overstated. These statements were based on the previously reported t-test. As we removed this analysis, these statements should be adjusted as well. Based on the interaction effect between touchxtimepoint and the steeper slope of CT-optimal touch in Fig 5, it appears that it is slightly more beneficial to use CT-optimal touch at the 15min timepoint compared to CT non-optimal touch. As it was also perceived as more pleasant and more feasible to apply for the partners, we do believe CT-optimal touch would be the preferable choice compared to CT non-optimal touch. We also added some information regarding the mere presence of partner as a form of passive social support at line numbers 508-509.

However, as mentioned this was still overstated so we changed this throughout the manuscript at line numbers: 21-22, 331-332, 390-392, 413-416, 476-486 and 522-526.

Line 481 “So, it could be that any form of affectionate touch might have similar effects”. This sentence is not understandable unless the authors want to say that only touch that is performed with CT-optimal velocity is affectionate? This claim seems to not be justified since other forms of touch such as hugs and handholding can certainly be perceived as affectionate. Do the authors mean “Any form of affectionate behaviour”, e.g. verbal support? This would make sense. Please reformulate.

As we indeed wanted to make this exact point, we reformulated this to affectionate behaviour at line number 507.

Line 482 “as there is an additional relieving effect of CT-optimal touch”. This is an overstatement, see comment further up. I would suggest to remove this sentence.

We removed this sentence and were more cautious with the other statements regarding the additional effect of CT-optimal touch. Also see the changes mentioned above.

Reviewer #2: All my coments have been addressed and the report has definitely been improved.

For future reference: the line numbers in the response letter did not match the manuscript (if I'm correct). I also think that the language can be improved here and there.

7. PLOS authors have the option to publish the peer review history of their article ([what does this mean?](https://journals.plos.org/plosone/s/editorial-and-peer-review-process#loc-peer-review-history)). If published, this will include your full peer review and any attached files.


If you choose “no”, your identity will remain anonymous but your review may still be made public.


**Do you want your identity to be public for this peer review?** For information about this choice, including consent withdrawal, please see our [Privacy Policy](https://www.plos.org/privacy-policy).

Reviewer #1: No

Reviewer #2: No
